# A potential prognostic biomarker SPC24 promotes tumorigenesis and metastasis in lung cancer

**DOI:** 10.18632/oncotarget.18971

**Published:** 2017-07-04

**Authors:** Juan Zhou, Yang Yu, Yunfeng Pei, Chunping Cao, Chen Ding, Duping Wang, Li Sun, Guoping Niu

**Affiliations:** ^1^ Department of Clinical Laboratory, Affiliated to Medical College of Southeast University and Xuzhou Central Hospital, Xuzhou, People’s Republic of China; ^2^ Department of Medical Oncology, Affiliated to Medical College of Southeast University and Xuzhou Central Hospital, Xuzhou, People’s Republic of China

**Keywords:** SPC24, lung cancer, prognostic biomarker, tumorigenesis, metastasis

## Abstract

**Results:**

SPC24 is over-expressed in clinical lung adenocarcinoma samples, and high level of *SPC24* is associated with advanced stages of lung tumors. Knocking down SPC24 repressed cell growth and promoted apoptosis. SPC24 deficiency reduced cancer cell migration as well. E-cadherin, one of the epithelial-mesenchymal transition markers, was up-regulated in the knockdown cells, along with down-regulation of N-cadherin and Vimentin. Oncomine expression analyses further confirmed that high level of SPC24 is associated with tumors from smokers, recurrent patients, or patients with shorter survivals.

**Purpose and methods:**

To reveal the role of SPC24, an important component of kinetochore, in the tumorigenesis of lung cancer, we performed Oncomine and immunohistochemistry (IHC) analyses for SPC24 in human lung adenocarcinoma tumors. We knocked down *SPC24* in two non-small cell lung cancer (NSCLC) cell lines, PC9 and A549, by siRNA and evaluated cell proliferation, apoptosis, and migration in the SPC24-deficient cells. Using a mouse xenograft model, we compared *in vivo* tumor growth of the knockdown and control cells. We further performed multiple Oncomine expression analyses for SPC24 in various lung cancer datasets with important clinical characteristics and risk factors, including survival, recurrence, and smoking status.

**Conclusions:**

*SPC24* is a novel oncogene of lung cancer, and can serve as a promising prognostic biomarker to differentiate lung tumors that have various clinicopathological characteristics. The findings of the current study will benefit the diagnosis, management, and targeted therapy of lung cancer.

## INTRODUCTION

As a leading cause of cancer deaths worldwide, lung cancer remains refractory among the major cancers. The five-year survival rate of lung cancer continues to be the lowest compared with other major cancers, such as colon, breast and prostate cancers [1]. Despite many therapeutic endeavors, the survival rate remains bleak, and staggers at about 15% five years after treatment [2]. The most frequent genetic alterations in lung cancer include mutations in *TP53*, *EGFR* (*Epidermal growth factor receptor*), *KRAS*, *EML4-ALK* (*echinoderm microtubule-associated protein-like 4-anaplastic lymphoma kinase*) rearrangements, and altered *MET* (*tyrosine-protein kinase Met*) signaling, which play critical roles in tumorigenesis of lung cancer, and therefore affecting the clinical sensitivity to targeted therapy [3, 4]. The fact that targeted therapy has been successful in a subset of tumors pleas for a better understanding of the pathological mechanisms by which these oncogenic alterations drive tumorigenesis of lung cancer [5]. Searching for novel molecular targets has thus become imperative for advancing targeted therapy of lung cancer.

SPC24 is a subunit of nuclear division cycle 80 (Ndc80) complex, a dumbbell-like, heterotetrameric structure in the outer kinetochore. Ndc80 is comprised of two heterodimers, CDCA1-KNTC2 and SPC24-SPC25. SPC24/SPC25 anchors the Ndc80 complex to the inner kinetochore, and mediates dynamic interactions between the nuclear spindle microtubules and kinetochores, ensuring faithful and accurate chromosomal segregation during mitosis [6, 7]. Therefore, dysregulation of any of these components in the kinetochore-microtubule interface can lead to genomic instability and disrupted control of cell cycle, which ultimately contribute to tumorigenic transformation.

The role of SPC24 in tumorigenesis awaits clarification. It has been reported that simultaneous disruption of both *SPC24* and *SPC25* genes renders the cell to be spindle checkpoint defective, which allows the cell to bypass mitosis in the absence of correct chromosomal segregation [8, 9], a process that resembles the features of the onset of tumorigenesis. High levels of CDCA1 (also known as NUF2, NDC80 kinetochore complex component), KNTC2 (kinetochore associated 2), SPC24, and SPC25 have been found to be correlated with colorectal and hepatocellular carcinoma tumors [10, 11]. Intriguingly, our initial investigation in Oncomine datasets indicated that SPC24 is up-regulated in lung adenocarcinoma tumors as well. Therefore, in the current study, we aimed at testing the hypothesis that SPC24 may promote tumorigenesis and progression of human lung cancer.

In this study, Oncomine analysis on various datasets and IHC staining in the clinical samples revealed up-regulation of SPC24 in human lung adenocarcinoma. We knocked down *SPC24* in multiple non-small cell lung cancer (NSCLC) cell lines by siRNA and evaluated cell proliferation, apoptosis, and migration of the *SPC24*-defective cells. We compared *in vivo* tumor growth of the knockdown and control cells in a mouse xenograft model. We also performed multiple Oncomine expression analyses for SPC24 in various lung cancer datasets with important clinical characteristics and risk factors, including staging, survival, recurrence, and smoking. Our results confirm the oncogenic role of SPC24 in the development and progression of lung cancer. Most importantly, SPC24 may serve as a promising prognostic biomarker for stratifying the lung cancer patients into clinically distinct groups that differ in staging, risk of smoking, recurrence, and survival.

## RESULTS

### SPC24 is over-expressed in human lung adenocarcinoma tumors

**Figure 1:**
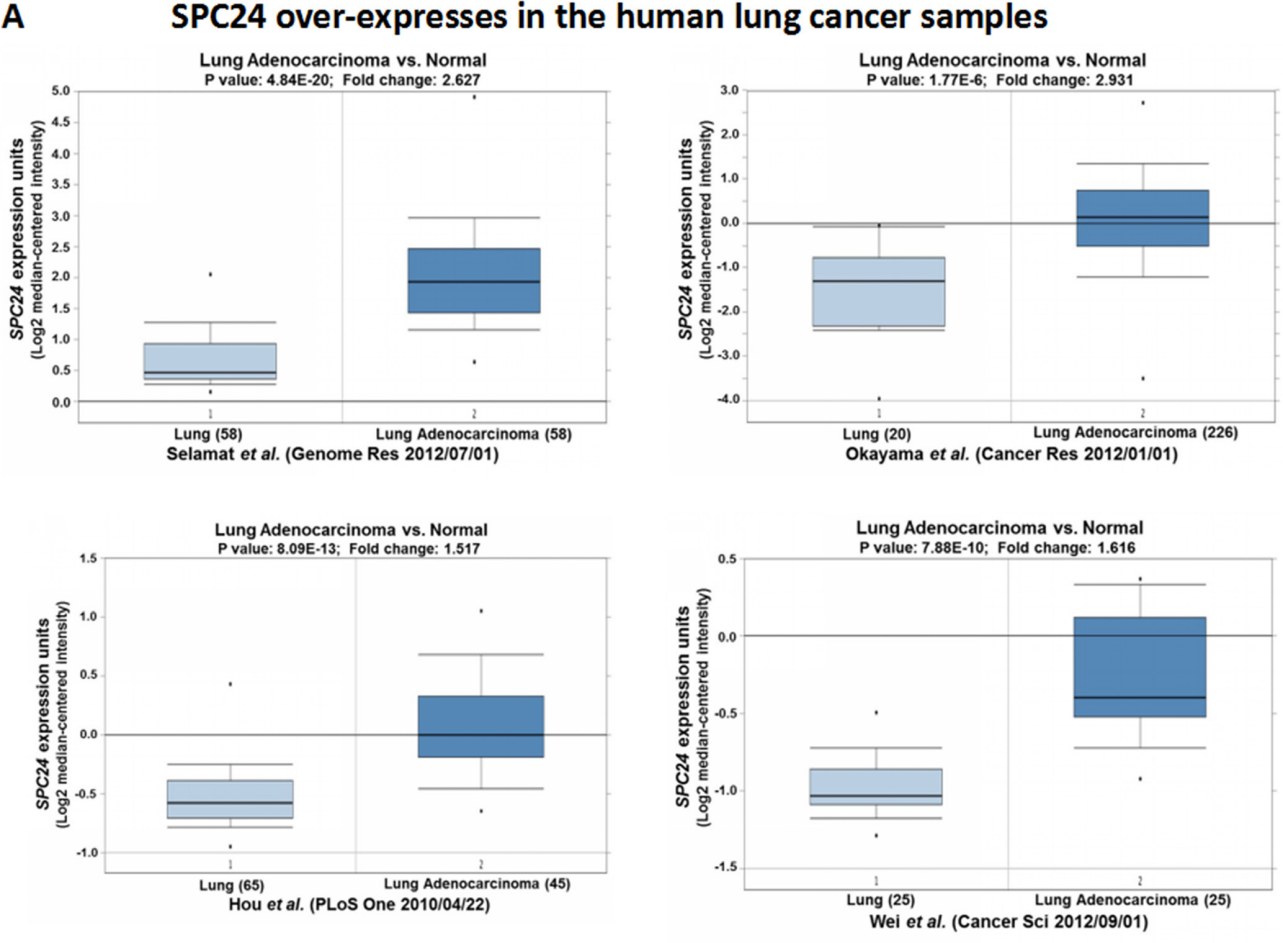

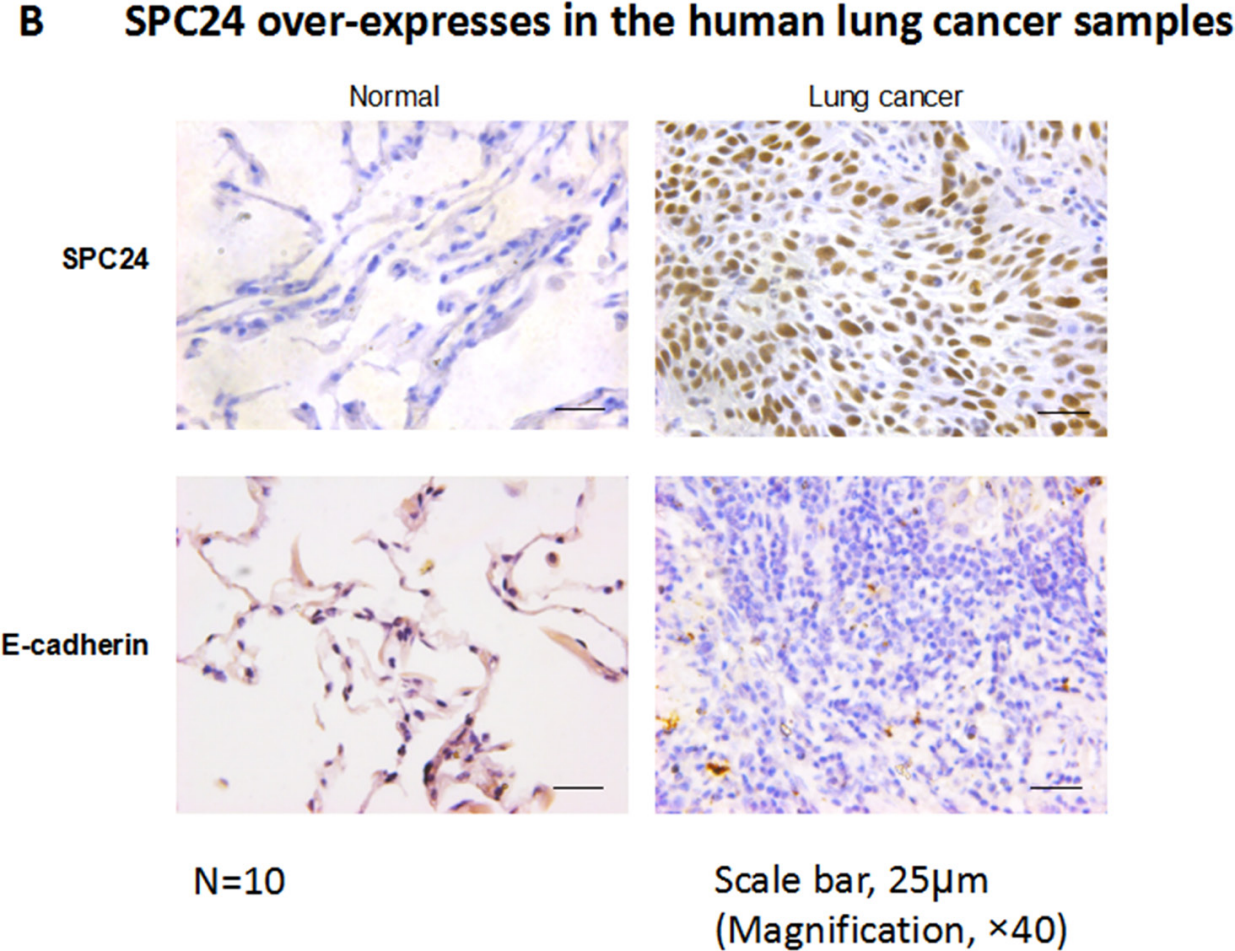

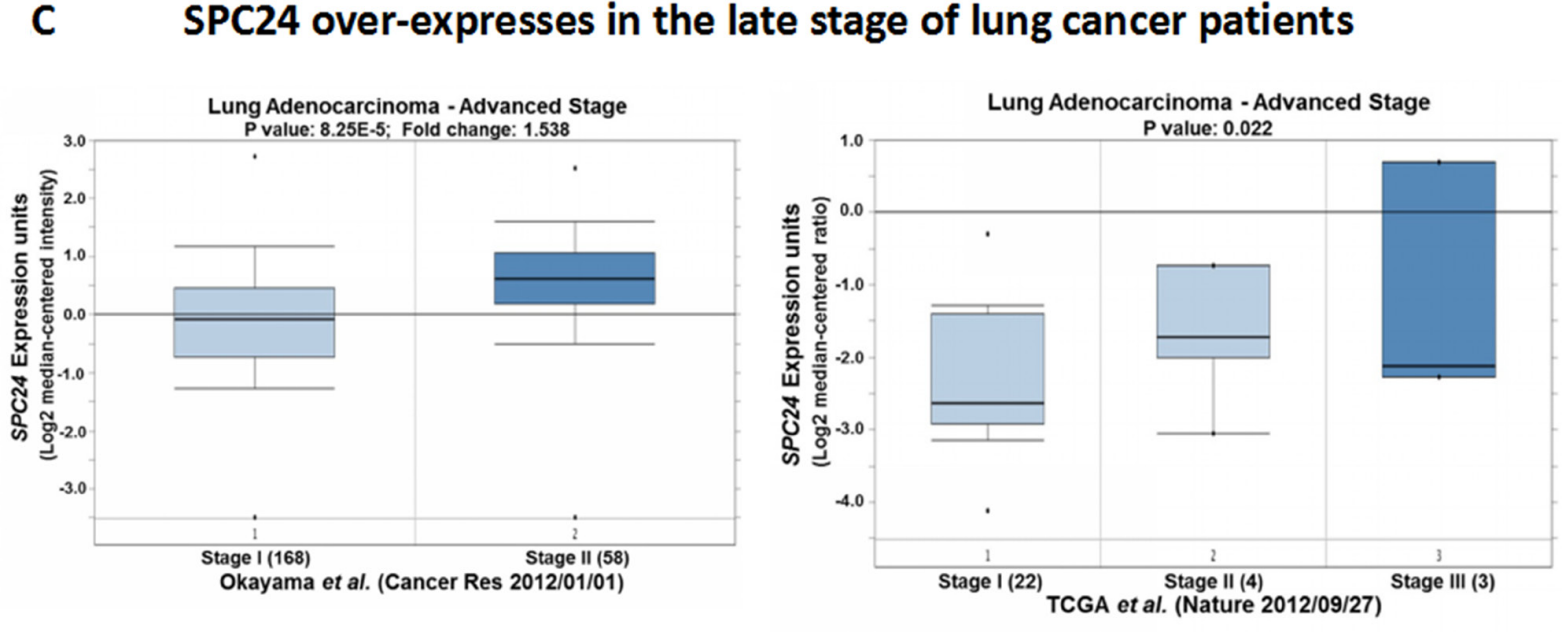
SPC24 is over-expressed in human lung adenocarcinoma tumors (**A**) Oncomine boxed plots of SPC24 in human adenocarcinoma and normal lungs. Oncomine data resources are listed under each plot. (**B**) IHC analysis of SPC24 in clinical normal and lung cancer samples, representing weak staining in the normal tissues, and strong one in the tumors. Ten (*N =* 10) tumors and 8 normal samples were examined. The staining signals are classified as weak, medium, and strong, and tabulated as the statistics for normal and tumor tissues. The χ^2^ statistic is 128.808, with *p-value* < 0.00001. Scale bar, 25 µm. (**C**) Oncomine boxed plots of SPC24 in lung adenocarcinomas of different stages (stage I, II, and III).

Previous work has suggested that disruption of *SPC24* renders the cell to be spindle checkpoint defective and confers uncontrolled mitosis [8]. High *SPC24* level has been reported for colorectal and hepatocellular carcinoma tumors [10, 11]. Therefore, we first performed Oncomine analysis in published datasets to examine the *SPC24* levels in human lung adenocarcinomas (Figure 1) [12, 13]. Interestingly, Oncomine boxed plot of *SPC24* expression levels between lung adenocarcinoma and normal samples in multiple datasets showed that *SPC24* is consistently over-expressed in lung adenocarcinomas (i.e. 1.5-fold or higher) compared with normal tissues (*p <* 10^–6^, Figure 1A) [14–17], indicating a positive role of *SPC24* in tumorigenesis of human lung cancer. To further confirm the Oncomine results, we performed IHC analysis in clinical lung tumor samples. Consistent with the Oncomine analysis, we found positive staining of *SPC24* is strongly correlated with tumors but not normal tissues (χ^2^ test, *p value* < 0.00001) (Figure 1B).

Based on these findings, we wondered if SPC24 levels could further stratify the lung adenocarcinoma tumors according to the staging. Interestingly, in one dataset, SPC24 levels are significantly higher in stage II adenocarcinoma tumors than stage I ones (*p <* 10^-4^, Figure 1C) [15]. Consistently, in the TCGA dataset, SPC24 levels are also significantly higher in tumors of advanced stages (stage II&III) compared with the stage I tumors (*p <* 0.05) [18]. The fact that SPC24 is over-expressed in lung adenocarcinoma, especially in those of the advanced stages, is suggestive of a promoting role of SPC24 in tumorigenesis of human lung cancer. Most importantly, it may also serve as a potential prognostic biomarker for stratifying patients into early and late stages of lung adenocarcinoma.

### Knocking down *SPC24* represses cell growth and promotes apoptosis in lung cancer cell lines

**Figure 2:**
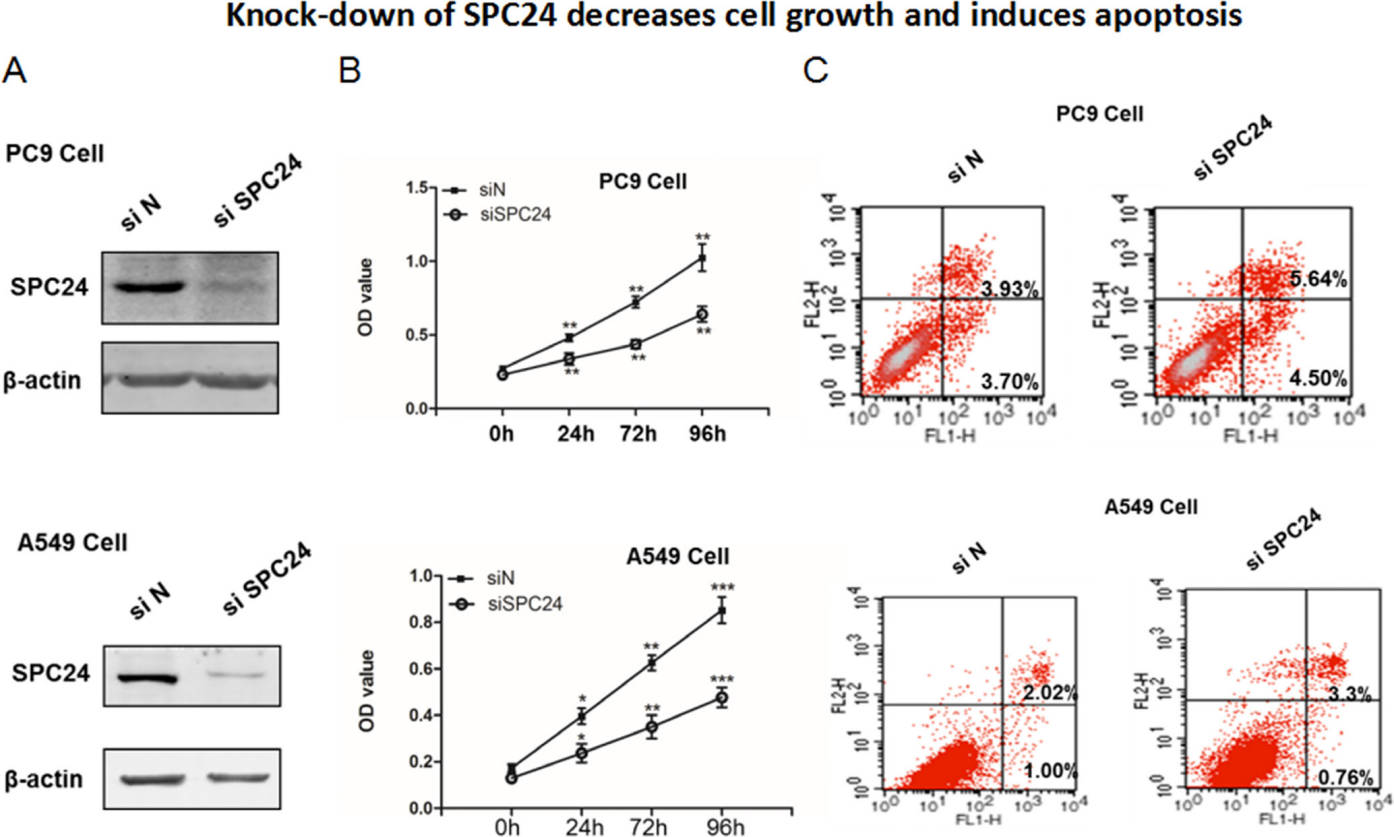
Knocking down *SPC24* represses cell growth and promotes apoptosis in lung cancer cell lines (**A**) *SPC24* was knocked down by siRNA in PC9 and A549 cells. Western blot of SPC24 was analyzed in the knockdown (si*SPC24*) and control (siN) cells. (**B**) Cell growth for *SPC24*-knockdown and control cells was measured as viable cell numbers that was recorded as OD_450_ at 0, 24, 72, and 96 h. (**C**) Apoptosis was recorded for knockdown and control cells by flow cytometry analysis of Annexin V-FITC-Propidium Iodide (PI) stained populations.

Dysregulated proliferation and apoptosis are the hallmarks of tumorigenicity of cancer cells. Since SPC24 is up-regulated in lung adenocarcinoma, we wondered if *SPC24* regulates cell growth and apoptosis in lung cancer. We knocked down *SPC24* in two non-small cell lung cancer (NSCLC) cell lines, PC9 and A549 (Figure 2), and measured proliferation and apoptosis in these cells. It is evident that the *SPC24*-knockdown (si*SPC24*) cells grew significantly slower than the control knockdown (siN) ones at all the time points tested (i.e. 24, 72, and 96 h). We also knocked down *SPC24* in an immortalized human bronchial epithelial cell line (BEAS 2B), and found that si-*SPC24* reduced cell growth with only marginal significance (*p <* 0.045) (Supplementary Figure 1). On the contrary, over-expression of SPC24 enhanced cell proliferation in both cancer cell lines (Supplementary Figures 2 and 3). Furthermore, flow cytometry analysis of apoptosis using Annexin V-FITC-PI staining showed that si*SPC24* cells had more apoptotic cells than siN ones. Notably, for PC9, the populations of both early stage (Annexin V ^+^/PI^−^) and late stage apoptotic (Annexin V ^+^/PI^+^) cells in si*SPC24* cells were higher than the siN control (Figure 2). Similarly, for A549, si*SPC24* had more apoptotic cells (Annexin V ^+^/PI^−^ and Annexin V ^+^/PI^+^ combined) than siN cells. Taken together, knocking down *SPC24* in NSCLC cells suppresses cell growth and promotes apoptosis, suggesting *SPC24* positively regulates cellular proliferation and viability in lung cancer.

### Knocking down *SPC24* suppresses cellular migration in lung cancer cell lines

**Figure 3:**
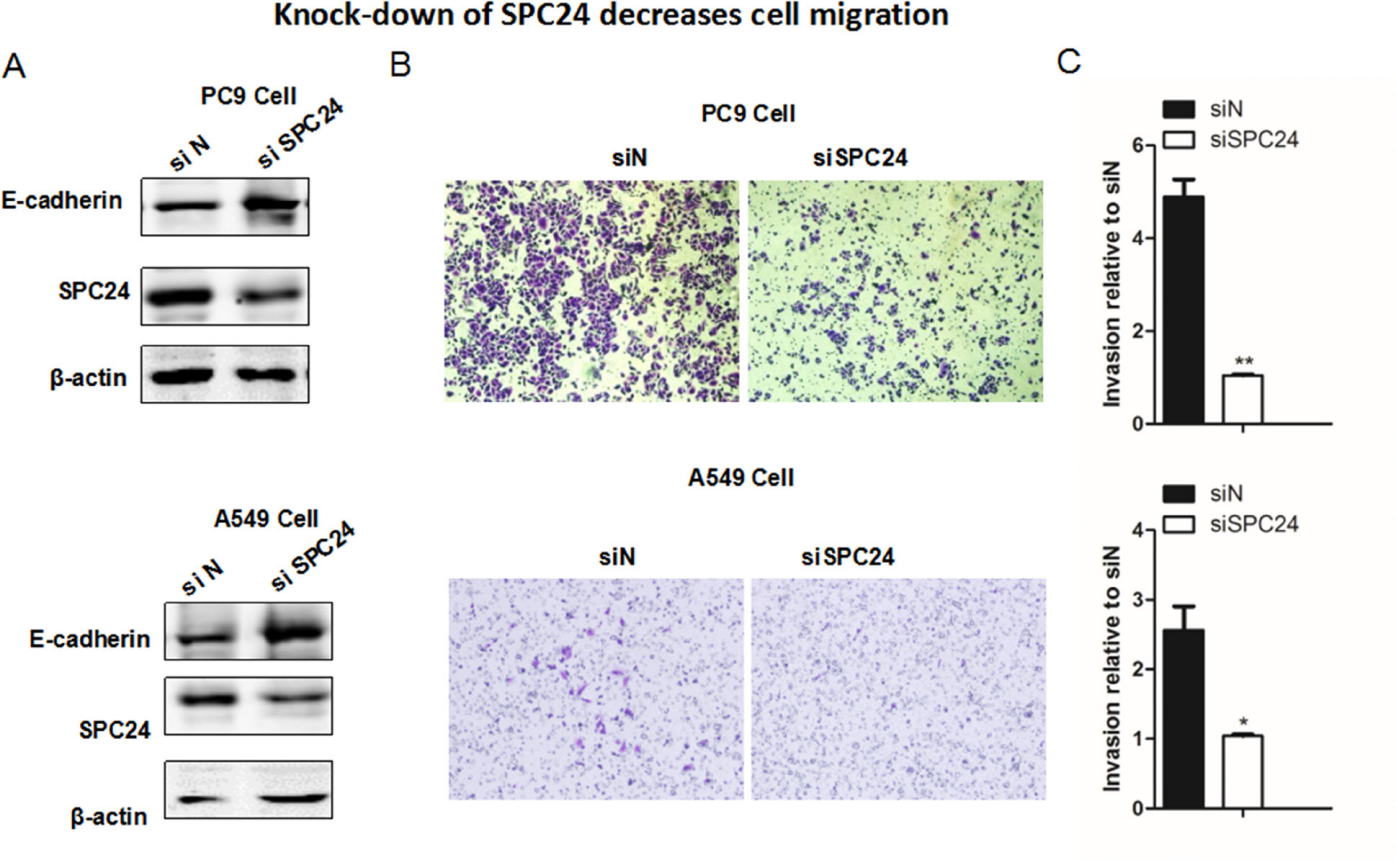
Knocking down *SPC24* suppresses cellular migration in lung cancer cell lines (**A**) Western blots of the EMT markers, including E-cadherin, Vimentin, N-cadherin,along with SPC24, are shown in SPC24-knockdown and control cells. (**B**) Migration of the knockdown and control cells was evaluated by Transwell migration assay. (**C**) Quantification of the cellular migration shown in (B). (Mean ± S.D., *n =* 3).

**Figure 4:**
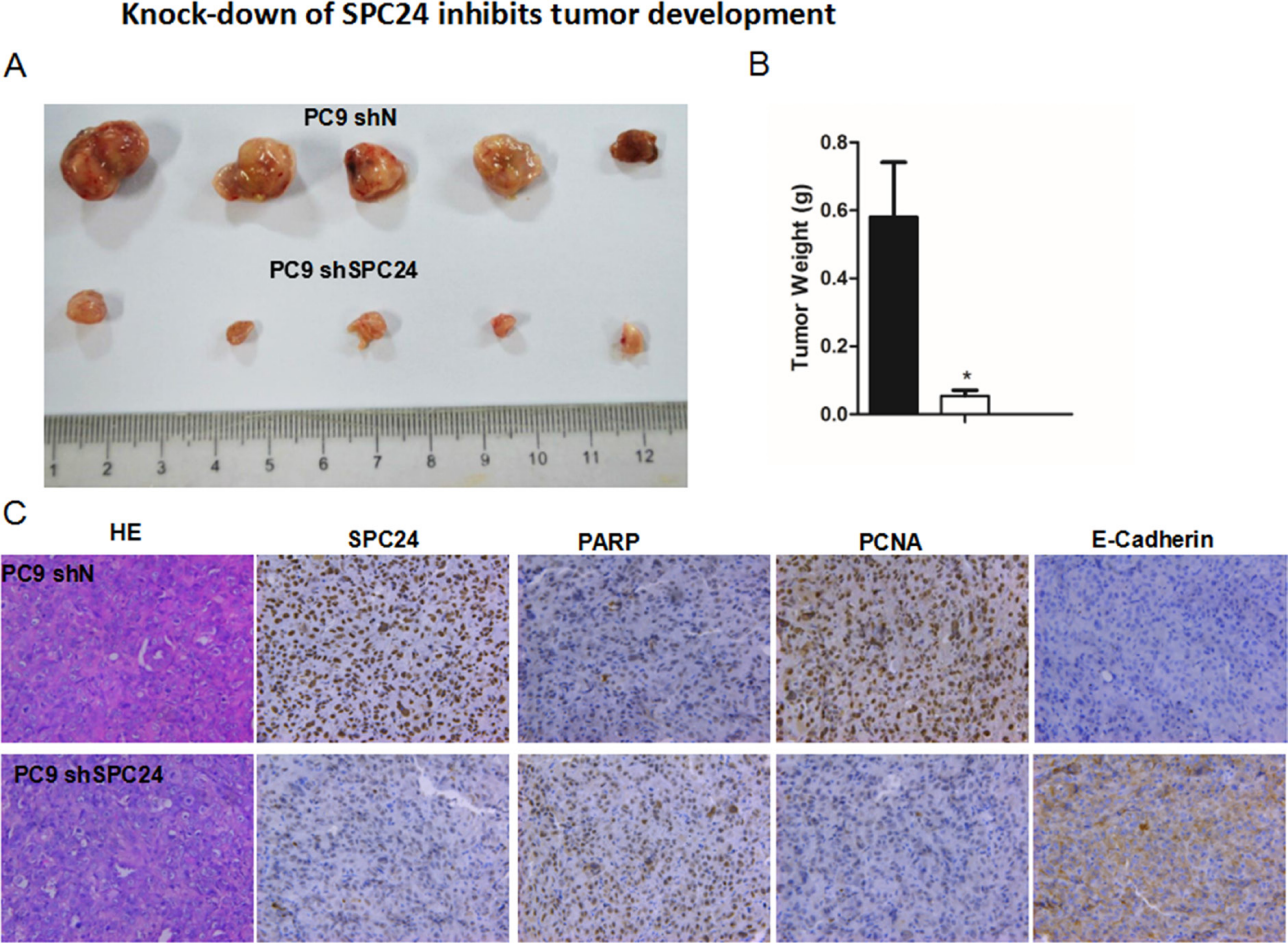
Knocking down SPC24 inhibits tumor growth *in vivo* (**A**) Stable *SPC24*- knockdown (sh*SPC24*) cell line of PC9 was generated by shRNA, and injected subcutaneously into 6-week old immunocompromised nude mice. At time of sacrifice, tumors were collected and weighed. (**B**) The weights of tumors are recorded as Mean ± S.D. (*n =* 3) *p < 0.05 (**C**) IHC staining of SPC24, PARP, PCNA, and E-cadherin was performed for SPC24-knockdown (shSPC24) or control (shN) tumors.Hematoxylin and eosin (HE) staining of the tumor tissues is also shown.

Given the result that *SPC24* positively regulates NSCLC cell proliferation, we further evaluated and compared the invasiveness of si*SPC24* and siN cells *in vitro*. Transwell migration assay showed that knockdown of *SPC24* significantly reduced migration of cells by 60% (A549, *p <* 0.05) to 80% (PC9, *p <* 0.01) (Figure 3), suggesting a promoting role of *SPC24* in lung cancer invasion. Most interestingly, we found E-cadherin level was up-regulated in si*SPC24* cells (Figure 3). Concomitantly, the levels of Vimentin and N-cadherin were considerably down-regulated in si*SPC24* cells, suggesting a promoting role of SPC24 in the regulation of epithelial-mesenchymal transition (EMT) for lung cancer. Consistently, over-expression of *SPC24* could enhance cell migration in both cancer cell lines (Supplementary Figures 2 and 3). This finding and the following IHC staining result of E-cadherin in mouse xenograft model (Figure 4) together support the notion that decreased *SPC24* leads to up-regulation of E-cadherin, marking a declined EMT process that is essential for metastasis. Furthermore, disruption of *SPC24* in the normal human bronchial epithelial cell line (BEAS 2B) did not affect cell migration, suggesting *SPC24* may play a more essential role in cancer progression than in the normal cell (Supplementary Figure 1). Therefore, our current data suggest that *SPC24* may positively regulate tumorigenic progression and invasion in NSCLC (see Discussion).

### Knocking down SPC24 inhibits tumor growth *in vivo*

The marked *in vitro* phenotypes observed for *SPC24* knockdown in the NSCLC cell lines prompted us to further evaluate its role in tumorigenic progression *in vivo*. We generated stable *SPC24*-knockdown cell line of PC9 (PC9 sh*SPC24*) and injected the sh*SPC24* or control (shN) cells into 6-week old immunocompromised nude mice, and recorded *in vivo* tumor growth for either sh*SPC24* or shN cells. At time of sacrifice, the average weight of sh*SPC24* tumors were significantly smaller than the shN tumors (*p <* 0.05), suggesting *SPC24* promotes tumorigenicity of lung cancer cells *in vivo* (Figure 4). IHC staining further showed that PARP (poly-ADP-ribose polymerase) and E-cadherin had been upregulated, yet PCNA (proliferating cell nuclear antigen) level downregulated, in the sh*SPC24* tumors, suggesting decreased *SPC24* may have resulted in DNA damage-induced hyperactivation of PARP [19], compromised cell proliferation [20], and decreased metastasis *in vivo*. Taken together, our data indicate that *SPC24* promotes NSCLC tumorigenicity both *in vitro* and *in vivo*.

### *SPC24* over-expression is observed in lung cancer patients who are smokers

**Figure 5:**
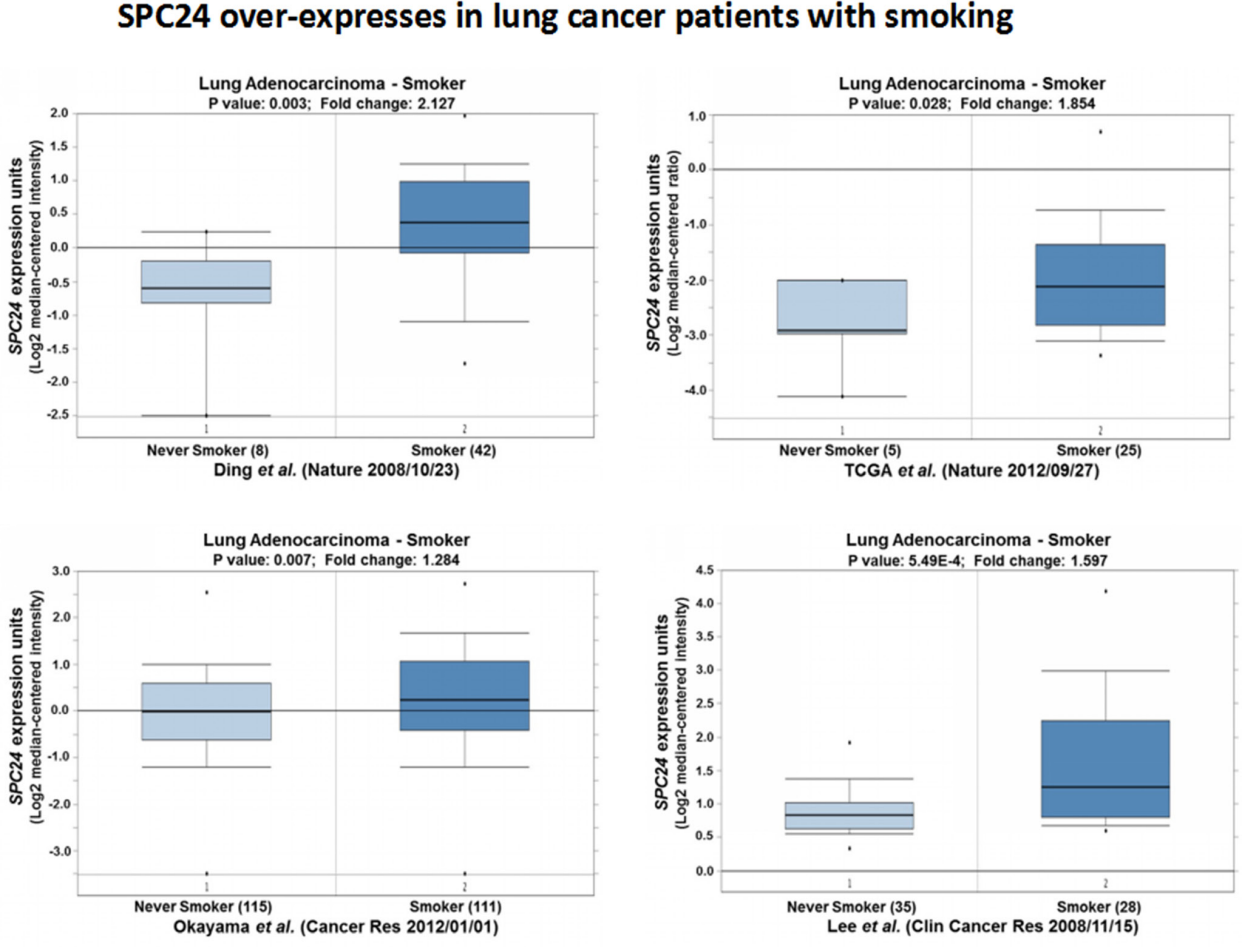
SPC24 over-expression is observed in lung tumors of whom the patients are smokers Oncomine boxed plots of SPC24 levels in lung adenocarcinomas from smokers vs. non-smokers. Four Oncomine expression analyses are shown.

Since smoking is the leading cause of small cell and non-small cell lung cancer, and contributes to more than 80 percent or more of lung cancer deaths, we further investigated the expression patterns of *SPC24* among the lung cancer patients who were smokers. Noticeably, we observed *SPC24* was significantly over-expressed in patients who were smokers in all four Oncomine expression datasets (*p <* 0.05, Figure 5) [15, 18, 21, 22]. Considering *SPC24* may promotes tumorigenesis of lung adenocarcinoma, this result suggests a possibility that *SPC24* may mediate smoking-induced tumorigenesis in lung cancer (see Discussion).

### *SPC24* over-expression is associated with lung cancer patients with recurrence or short survivals

**Figure 6:**
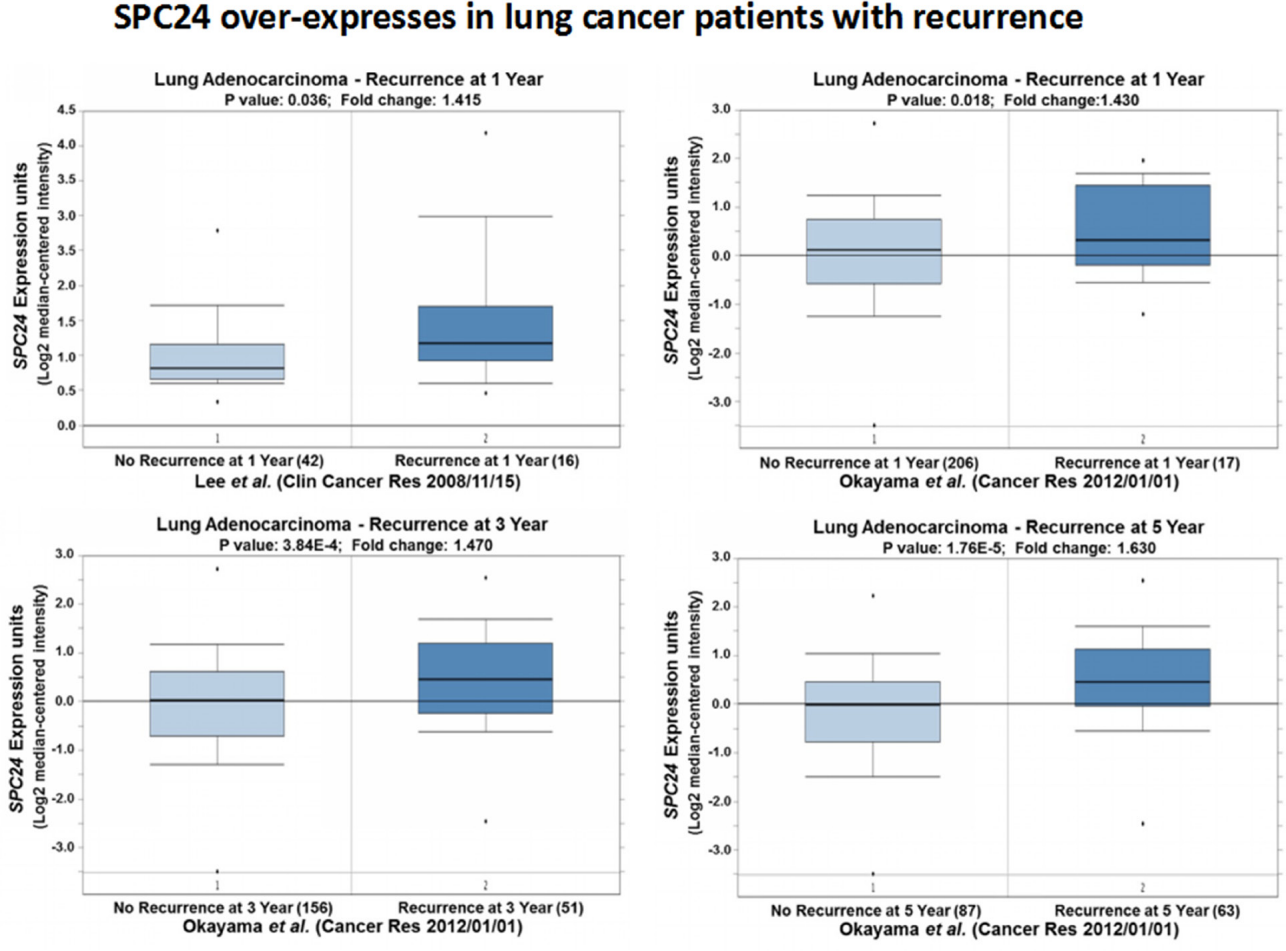
SPC24 over-expression is associated with lung tumors from patients with recurrence Oncomine boxed plots of SPC24 levels in lung adenocarcinomas from patients with or without recurrent cancer. Four Oncomine expression analyses are shown.

So far, both the *in vitro* and *in vivo* data suggest *SPC24* promotes tumorigenesis of lung cancer. Furthermore, we also observed significantly high levels of *SPC24* in lung adenocarcinoma, especially the advanced stage, tumors. These results indicate SPC24 is a promising prognostic biomarker for lung cancer. We went forward to decide if SPC24 could serve as a potential biomarker for predicting the prognosis of patients who had either recurrent lung cancer or short survivals. The Oncomine expression analysis revealed that *SPC24* was over-expressed in tumors from patients who had recurrent cancer after one year (*p <* 0.05, Figure 6) [22]. On the other hand, another Oncomine expression analysis performed on a more complete dataset showed that *SPC24* was consistently over-expressed in tumors from patients who regained the cancer after one, three, or five years, compared with those who had not suffered recurrence. Most importantly, the later the patients had the recurrent cancer, the higher *SPC24* levels were observed, and the trend became statistically significant along the timeline (Figure 6) [15].

**Figure 7:**
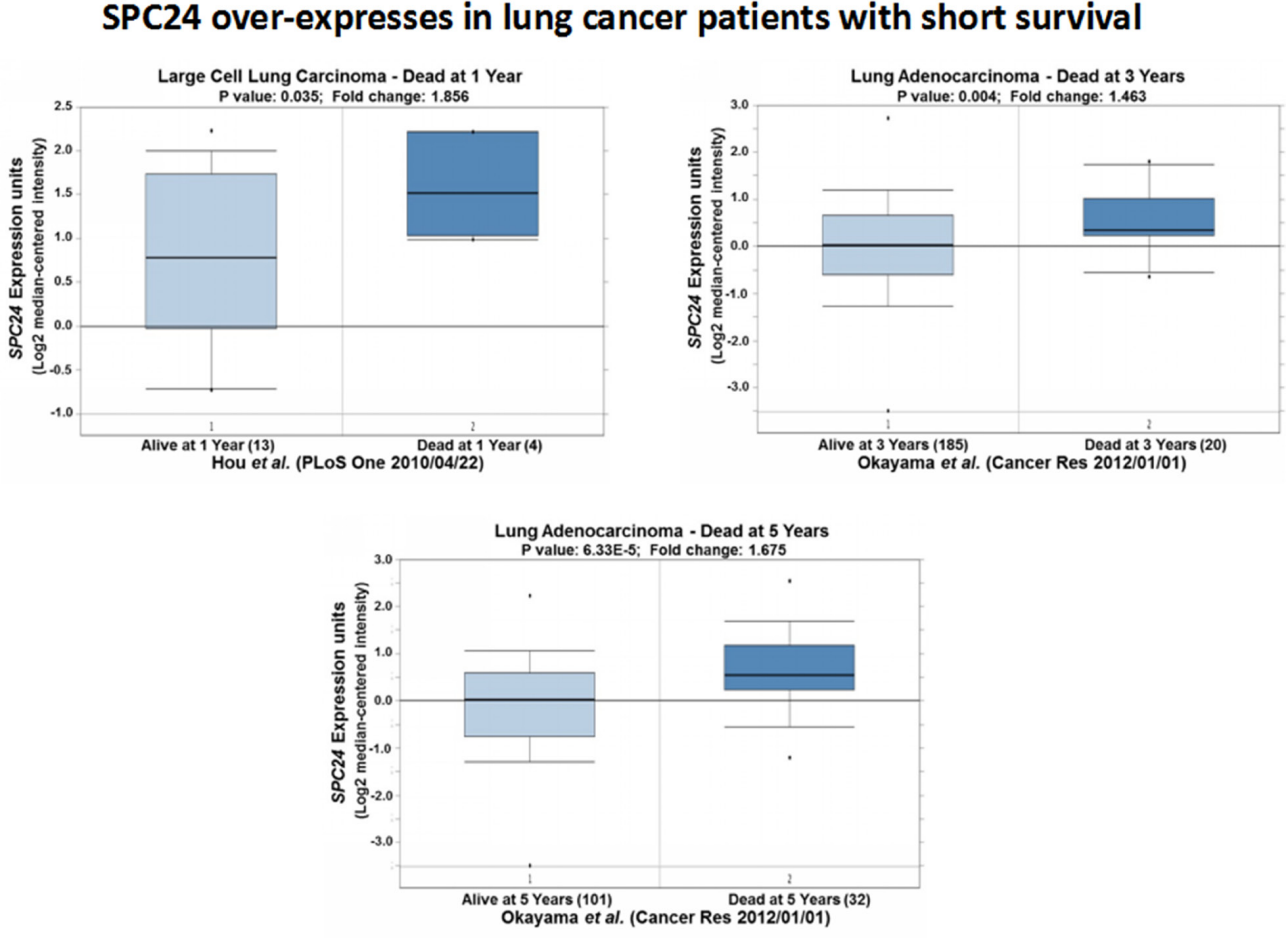
SPC24 over-expression is associated with lung tumors from patients with short survival Oncomine boxed plots of SPC24 levels in lung adenocarcinomas from patients who died at 1, 3, or 5 years after diagnosis, compared with those who were still alive at the same time point.

Further Oncomine expression analyses on the lung cancer patients with short survivals recapitulated the over-expression pattern of *SPC24* in the lung tumors: among the patients who died after one year, *SPC24* levels were the highest compared with those who died three or five years later (Figure 7) [14, 15]. This result, along with the one obtained for recurrent tumors, suggests that SPC24 can serve as an efficient prognostic marker for lung cancer in the clinic.

## DISCUSSION

In the current study, to the best of our knowledge, we for the first time provide evidence to support the oncogenic role of SPC24 in lung cancer development and progression. SPC24 is over-expressed in human lung adenocarcinoma tumors. Knocking down *SPC24* in NSCLC cells significantly suppressed cellular proliferation and promoted apoptosis. *SPC24*-knockdown cells also displayed significantly compromised invasion in Transwell migration assay. In accordance with this phenotype, up-regulation of E-cadherin and down-regulation of N-cadherin and vimentin were observed in si*SPC24* cells, suggesting that SPC24 may positively regulate EMT and metastasis in NSCLC. *In vivo*, *SPC24*-knockdown cells formed much smaller tumors compared to the unmodified control cells in the mouse xenograft model of NSCLC. Most importantly, multiple Oncomine expression analyses of *SPC24* confirmed that high level of *SPC24* is associated with lung tumors from smokers, recurrent patients, and patients with shorter survivals.

At the interface of the nuclear spindle microtubules and kinetochores, spindle machineries essentially control and ensure faithful chromosomal segregation. Disruption of spindle checkpoint proteins leads to chromosomal missegregation and alteration in chromosome numbers in the daughter cells, eventually causing massive genetic instability and aneuploidy, a hallmark of cancer [23]. In this study, we show direct evidence that down-regulation of SPC24, a key component linking inner and outer kinetochore, can reduce proliferation of lung cancer cells. The decreased viability is concomitant with increased apoptosis in *SPC24*-knockdown cells, suggesting SPC24 is essential for the survival of the cancer cells (Figure 2). Consistent with the *in vitro* phenotypes, the ability of the *SPC2*4-knockdown cells to form tumors is significantly reduced, further attesting the importance of maintaining the integrity of the spindle machineries to promote tumorigenicity of lung cancer cells. IHC analysis on the xenograft tumors also revealed a decreased level of PCNA, a marker of cell proliferation, suggesting the viability of the *SPC24*-knockdown cells had been compromised *in vivo*. Oncomine expression analysis show that *SPC24* is over-expressed in lung adenocarcinoma tumors, providing another layer of evidence that SPC24 positively regulates lung cancer development.

Supporting the notion that SPC24 may positively regulate metastasis of lung cancer, *SPC24*-knockdown cells display markedly decreased migration and invasion. EMT markers, such as E-cadherin, is up-regulated, along with down-regulation of N-cadherin and vimentin (Figure 3), suggesting that SPC24 may promote EMT, a hallmark of metastasis [24]. As a side note, down-regulation of E-cadherin has been implicated in invasive NSCLC in the clinic [25]. Thus, based on the results from the *in vitro* proliferation and invasion assays and the *in vivo* tumorigenicity assay, we conclude that SPC24 is a novel oncogene of lung cancer.

Consistent with this conclusion, we show that up-regulation of *SPC24* is generally associated with poor prognosis of lung cancer patients. Up-regulation of *SPC24* is associated with advanced lung adenocarcinoma tumors, and tumors from smokers. More importantly, patients with recurrent cancer or short survivals have higher levels of SPC24. Therefore, SPC24 is a potential prognostic marker of NSCLC. Previous work has suggested that standalone markers, such as p53, p21, Ki-67, PCNA, KRAS, and cyclin D1, cannot serve as efficient prognostic markers for NSCLC due to NSCLC heterogeneity [26–28]. Instead, combinatorial use of these markers will perform better for prognostic prediction, and thus is more clinically meaningful [26, 27, 29].Our current work suggests that SPC24 can serve as a potentially efficient marker for staging the lung tumors and predicting the clinical prognosis of the patients. As a result, it is anticipated that SPC24 would complement the currently available markers for prognostic prediction of NSCLC in the clinic.

In summary, our study reveals a new link between chromosomal missegregation-induced genetic instability and tumorigenesis of lung cancer. In light of the discovery, future studies should be directed to (1) interrogate the mechanism by which SPC24 stimulates development of lung cancer; (2) reveal how SPC24 promotes EMT and metastasis; (3) validate SPC24 as a prognostic marker of lung cancer in clinical cohorts; and (4) test if other components of the spindle checkpoint machinery will confer similar functions as what has been observed for SPC24. From the clinical perspective, confirmation of SPC24 as a prognostic biomarker can benefit the diagnosis, management, and targeted therapy of lung cancer.

## MATERIALS AND METHODS

### Animals

Female mice were purchased from Shanghai Laboratory Animal Co. Ltd. (SLAC, Shanghai, China), and maintained regularly with readily accessible food and water. All animal procedures were approved by the Institutional Bioethics Committee and complied with the regulations on animal welfare.

### Cell culture

Human non-small cell lung adenocarcinoma cell lines PC9 and A549 were obtained from ATCC. Both cell lines were cultured in RPMI 1640 medium supplemented with 2 mM L-glutamine, 10% fetal bovine serum (FBS), 100 U/ml penicillin, and 100 mg/ml streptomycin. The cell cultures were maintained at 37°C under a humidified atmosphere consisting of 95% air and 5% CO_2_.

### RNA interference of SPC24

*SPC24* small interference RNAs (siRNAs) were selected based on the program on http://jura.wi.mit.edu/bioc/siRNAext/ and synthesized at Shanghai GenePharma Co. The siRNA sequences are 5′-GAGCCUUCUCAAUGCGAAGTT-3′ and 5′-CUUCGCAUUGAGAAGGCUCTT-3′, and the following scrambled siRNA was used as the control: 5′-GAGUUAAAGUCAAAGUGACTT-3′ and 5′-GUCACUUUGACUUUAACUCTT-3′.To exclude the off-target sequences, BLAST search was performed against the human genome database and the above sequence was confirmed to be *SPC24*-specific.

### Proliferation assay

Cell proliferation assay was performed according to the manufacturer’s manual (Cell Counting Kit-8, Dojindo Molecular Technologies, Inc., USA), based on reduction of a tetrazolium salt into a water-soluble formazan dye in a living cell. Both targeted-knockdown (si*SPC24*) or knockdown control (siN) cells (1 × 10^5^ cells/well) were cultured for 0, 24, 72, 96 h, the absorbance at 450 nm at each time point was assayed.

### Analysis of apoptosis

Analysis of apoptosis is based on Annexin V-FITC-Propidium iodide (PI) staining method. Briefly, cells were harvested and washed by cold PBS at a density of 1 × 10^6^ cells/mL. A mixture of PI (final concentration 100 µg/mL) and Annexin V-Alexa Fluor488 conjugate was then incubated with the cells at room temperature for 15 min. Flow cytometry was used to analyze apoptosis with the parameters of 494/518 nm set for Annexin V dectection and 535/617 nm for PI.

### Cellular migration assay

A transwell insert (Falcon 354480; BD Biosciences) was used for cellular migration assay. Briefly, SPC24-knockdown and scramble iRNA controls cells were harvested and re-suspended with serum-free DMEM medium at a concentration of 1 × 10^5^ cells/mL. Cell suspension (100 μL) was added to the upper chamber. The lower chamber was loaded with 600 μL of DMEM medium supplemented with 20% FBS. In control experiments, medium containing 1% FBS was added to the lower chamber. The unit was incubated at 37°C for 20 hr. Cells migrating through the membrane to the bottom chamber were then fixed by 4% paraformaldehyde and stained with 0.5% crystal violet for 20 min. Six microscopic fields (magnification, × 100) were randomly photographed for cell counting. Each experiment was repeated three times for the calculation of the means and standard deviations.

### Mouse xenograft tumor model

Briefly, *SPC24*-knockdown and control cells were harvested and suspended at a concentration of 5 × 10^5^ cells/100 µL in cold PBS. Freshly prepared cells were injected into the nude mice subcutaneously. Mice were examined twice a week for 10 weeks where tumor size and weight loss were assessed and recorded. Mice were euthanized by CO_2_ asphyxiation when they lost more than 20% of their body weight before injection. Tumors were then removed at the time of sacrifice, embedded in paraffin, sectioned, and stained with hematoxylin and eosin (H&E) or immunohistochemistry (IHC).

### Western blot

For Western blot, nuclear extracts were separated on 12% SDS-polyacrylamide gels and transferred to polyvinylidene difluoride (PVDF) membranes. Membranes were blocked in 5% non-fat milk in Tris-buffered saline (plus 0.1% Tween-20). SPC24 (Cat. ab169786), E-cadherin (Cat. ab1416), and β-actin (Cat. ab8226) antibodies were purchased from Abcam. Blots were detected by enhanced chemiluminescence and exposed on X-ray films.

### Immunohistochemistry (IHC)

Continuous sections of tumor or normal tissues were prepared from paraffin-embedded blocks. After deparaffinization in xylene, the sections were rehydrated in a graded ethanol series. Antigen retrieval was performed by microwaving for 3 min in citrate-buffered solution (pH6.0). Endogenous peroxidase activity was quenched by incubation in 3% hydrogen peroxide for 20 min. Blocking was done by pre-incubation with 10% goat serum at room temperature for 30 min. Sections were incubated with antibodies indicated in this study (i.e. SPC24 [rabbit Ab 1:200 dilution Abcam]), PARP, PCNA, E-Cadherin) for overnight in a humidified container at 4°C. The following day, after PBS washes, incubation with the secondary antibody conjugated with horseradish peroxidase was performed for 1 hr at room temperature. Sections were finally stained with 3, 3- diaminobenzidine tetrahydrochloride (DAB) and counterstained with hematoxylin. A negative control was obtained by replacing the primary antibody with normal rabbit serum.

### Statistical analysis

Student’s *t* tests were performed to analyze *in vitro* data. For mouse studies, two-tailed *t* test was used to compare tumor weights between control and SPC24-knockdown groups. *P value*s < 0.05 were considered significant.

## SUPPLEMENTARY MATERIALS FIGURES



## Abbreviations

IHC: Immunohistochemistry
NSCLC: non-small cell lung cancer
EGFR: Epidermal growth factor receptor
EML4-ALK: echinoderm microtubule-associated protein-like 4-anaplastic lymphoma kinase
MET: tyrosine-protein kinase Met
Ndc80: nuclear division cycle 80
KNTC2: kinetochore associated 2
FBS: fetal bovine serum
siRNAs: small interference RNAs
H&E: hematoxylin and eosin
PVDF: polyvinylidene difluoride
DAB: 3, 3- diaminobenzidine tetrahydrochloride
