# Supplementary material for: A potential prognostic biomarker SPC24 promotes tumorigenesis and metastasis in lung cancer
Source: Oncotarget. 2017 Jul 4;8(39):65469–80. doi: 10.18632/oncotarget.18971 (PMC5630346; doi:10.18632/oncotarget.18971)
Supplement: Supplementary file 1 [file oncotarget-08-65469-s001.pdf]

A potential prognostic biomarker SPC24 promotes tumorigenesis and metastasis in lung cancer

Supplementary Materials

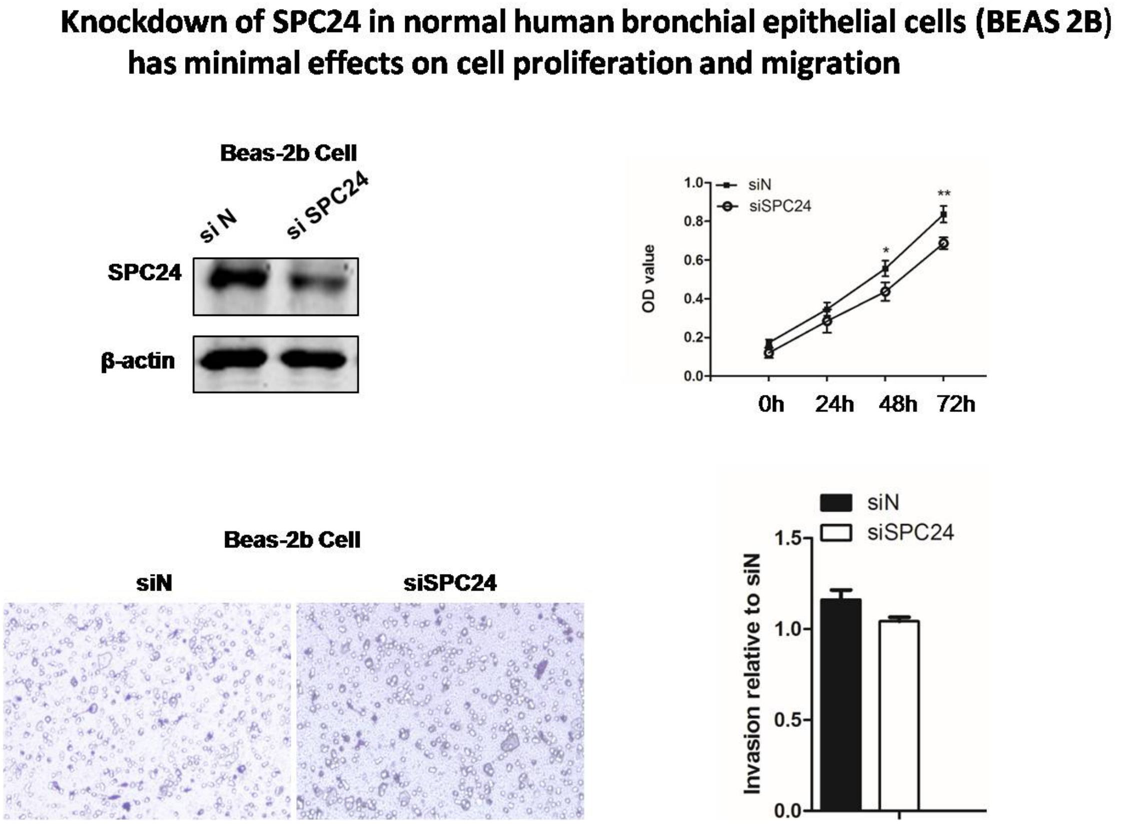

Supplementary Figure 1: Knockdown of SPC24 in normal human bronchial epithelial cells (BEAS 2B) has minimal effects on cell proliferation and migration. *SPC24* was knocked down by siRNA in BEAS 2B cells. Cell proliferation for *SPC24*-knockdown and control cells was measured as viable cell numbers that was recorded as OD_{450} at 0, 24, 72, and 96 h. Transwell cell migration was also assayed and quantified for the control and si*SPC24* cells.

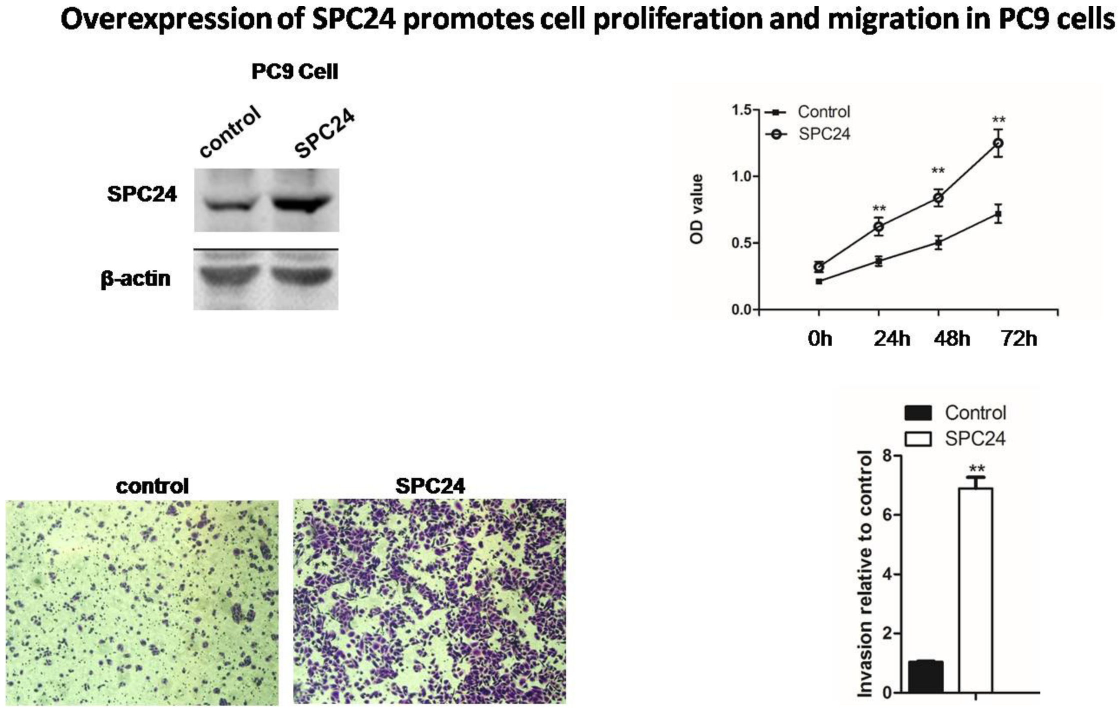

Supplementary Figure 2: Over-expression of SPC24 promotes cell proliferation and migration in PC9 cells. SPC24 was over-expressed in PC9 cells. Cell proliferation and migrations were measured by the OD_{450} colorimetric and Transwell assays.

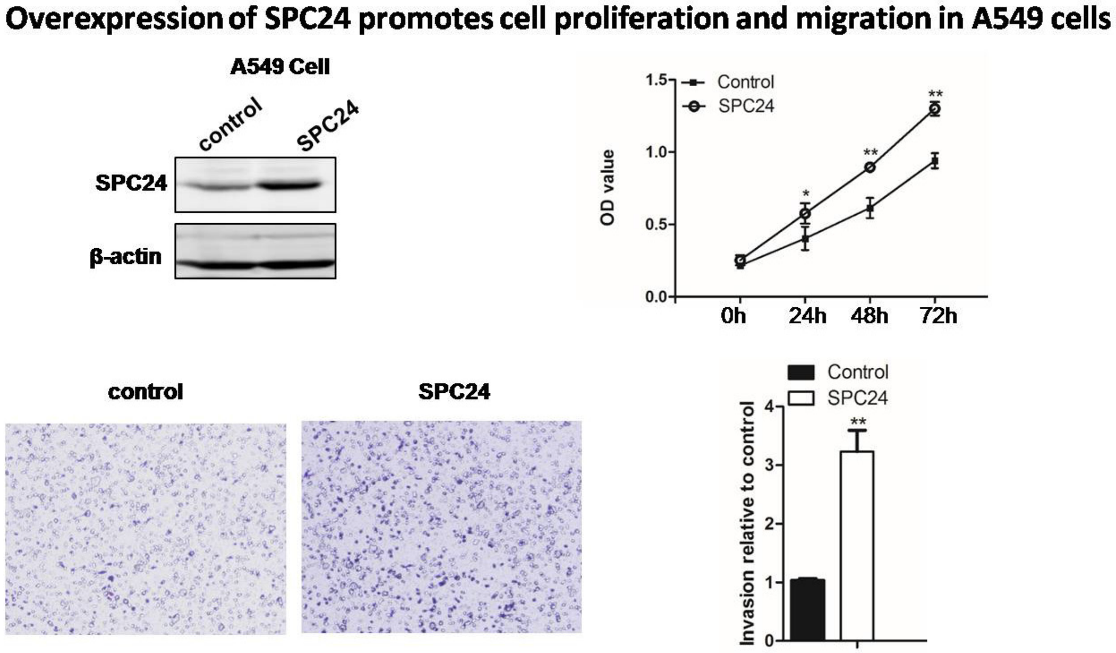

Supplementary Figure 3: Over-expression of SPC24 promotes cell proliferation and migration in A549 cells. SPC24 was over-expressed in A549 cells. Cell proliferation and migrations were measured by the OD_{450} colorimetric and Transwell assays.
